# Silk Fibroin Protective Coating for Washable and Reusable Textile Electronics

**DOI:** 10.3390/ijms26209848

**Published:** 2025-10-10

**Authors:** Anna Baranowska-Korczyc, Dorota Kowalczyk, Małgorzata Cieślak

**Affiliations:** Department of Chemical Textiles Technologies, Łukasiewicz Research Network—Lodz Institute of Technology, 9/27 M. Skłodowskiej-Curie Street, 90-570 Lodz, Poland

**Keywords:** silk fabric, silk fibroin, SWNTs, HiPCO, textile electronics

## Abstract

In this study, a new way of protecting textile wearable electronics is proposed. A natural product, silk fibroin, known for its high biocompatibility, biodegradability, and low cytotoxicity, was selected to cover the functionalized fabric to improve its stability and enable washability. Silk fabric was selected as a non-toxic material, suitable for further application on skin and for wearable devices. Silk fabric was functionalized with various amounts of high-pressure carbon monoxide single-walled carbon nanotubes (HiPCO SWNTs). HiPCO SWNTs made the fabric electroconductive, but they are easily washed out of the fabric. The fabric functionalized with HiPCO SWNTs was covered with silk fibroin (SF) protein, which was subsequently crystallized by ethanol vapor to make it insoluble in water. The functionalization and silk fibroin coverage processes were studied using electrical resistance measurements, infrared and Raman spectroscopies, thermogravimetric technique, and surface wettability analysis. The coverage of the fabric with crystallized silk fibroin enables the washing process. The resistance of the functionalized fabric with silk fibroin did not increase significantly. The presented silk fibroin coating can facilitate the construction of future wearable electronics, protect the electroconductive nanomaterials on the fabric surface, and make textile structures reusable.

## 1. Introduction

In recent years, wearable textile electronics have gained attention due to their advancements in remote health monitoring, medical diagnosis and treatment, as well as the generation and storage of energy [1]. The market for e-textiles has been growing rapidly and is predicted to grow to $5 billion by 2027, and the growth is forecasted to be more than $1.3 billion per year by 2031 [2].

Various types of fibers have been utilized in designing smart textiles, including synthetic, conductive carbon, conductive polymers, and electrospun nanofibers [1,3]. One of the most valuable materials in soft electronics is silk fabric, which acts as a sustainable platform for flexible, wearable, and biodegradable electronics [4]. Silk fabric is made of silk filament fibers, which contain silk fibroin, characterized by high mechanical strength, high biocompatibility, tunable degradation rate, low cytotoxicity, and optical transparency, making it a robust substitute for synthetic materials in wearable textile electronics [4]. The key issue in designing smart textiles is making them conductive. Conductive particles can be added directly into polymer yarns or combined with conductive materials, including metals or conductive polymers. It is also possible to combine polymers with inorganic nanomaterials and make conductive hybrids. Surface functionalization is also an efficient method for improving the electrical conductivity of textile structures. Silk structures are made conductive mainly by coating or doping with conductive materials, nanoparticles, conductive polymers, or carbon-based nanomaterials [1,3].

Natural glue–sericin was degummed by sodium carbonate 10%wt. aqueous solution at about 80 °C and replaced by MXene/sodium alginate layer. The ultrathin sheath layer was tightly bridged with the core silk filament fiber made of silk fibroin through strong interfacial interactions, which resulted in high electroconductivity and tensile strength, and a formation tool for monitoring human pulse, body movements, and changes of ambient humidity in real time [5].

A wearable electronic skin system was proposed on a composite ionic conductive hydrogel made of silk fibroin and polyacrylamide [6]. Ti3C2Tx-silver@silk nanofiber composites with multi-dimensional heterogeneous conductive networks were synthesized using combined in situ growth and vacuum filtration methods and applied to recognize human gestures [7]. Silk fibroin nanofiber mats coated with polypyrrole, characterized by high conductivity, were prepared through electrospinning and chemical polymerization for potential use in peripheral nerve regeneration [8]. Functionalization of silk fabric with graphene makes it electroconductive, antibacterial, and heat-protective [9]. The selection of silk fibroin enables the design and creation of wearable electronics without environmental or toxic concerns for humans [10].

The selected functionalization or integration of conductive materials with silk fibroin should be stable and ensure reusability. There are various approaches to support the stability of modified silk fibroin. One of them is in situ synthesis of inorganic particles, e.g., platinum nanoparticles can be formed directly on the silk surface through the reduction of Pt ions using heat treatment [11]. This method created a stable coating with excellent wash and rub fastness, providing catalytic and antibacterial properties. Dip-coating with adhesion promoters, including polydopamine, is also an efficient method. Silk fabric was functionalized with polydopamine and further modified with silver nanowires [12] and single-walled carbon nanotube-based ink [13] to make it both electroconductive and washable. The other strategy is to combine silk fibroin with poly(3,4-ethylenedioxythiophene) poly(styrene sulfonate) (PEDOT:PSS) for efficient interfaces between electronic systems and biological environments [14], and to develop methods for enhanced transdermal delivery performance [15].

In this research, silk fabric was protected after dip-coating with conductive nanomaterial by forming a silk fibroin coating. To the best of our knowledge, silk fibroin as a protective layer was not applied for textile electronics, only for food preservation [16], seed coating [17], and medical implants [18]. Silk fibroin was applied in this study in two ways: as a model textile structure for functionalization with conducive nanomaterials, and as a protective cover for conductive functionalized fabric. Silk fabric was covered with various amounts of high-pressure carbon monoxide single-walled carbon nanotubes (HiPCO SWNTs). It renders silk fabric electroconductive, suitable for wearable electronics, but not washable or suitable for repeated use. For these purposes, silk fabric functionalized with HiPCO was coated with silk fibroin, which was subsequently crystallized by ethanol vapor to obtain its stable, water-insoluble β-sheet form. This new approach enables the creation of electroconductive, stable, and washable silk fibroin-based materials for designing devices in wearable electronics.

## 2. Results and Discussion

In this study, silk fabric was modified with HiPCO SWNTs. The synthesis reaction of HiPCO uses metal carbonyls as a catalyst, along with high temperature (~1000 °C) and high pressure (~100 atmospheres) to produce SWNTs of small diameter and high purity [19]. HiPCO SWNTs serve as a benchmark for both the academic community and industrial and commercial applications [19]. Silk fabric was labeled as fabric, HiPCO SWNTs as HiPCO, and silk fabric after the functionalization with nanotubes as fabric_HiPCO0.01, fabric_HiPCO0.03, fabric_HiPCO0.05, or fabric_HiPCO0.06, depending on the deposited HiPCO amount on the fabric. The characteristics of the sample and details of their functionalization process are presented in Table 1. Ten separate samples were taken to determine each characteristic. After HiPCO deposition on silk fabric, they were covered with silk fibroin (SF) to protect HiPCO and to make them washable and reusable. To make silk fibroin insoluble in water, the protein was induced to form a crystalline structure by ethanol vapor, according to the procedure presented in our previous report [12].

The samples covered with SF were labeled as fabric_HiPCO0.01/SF, fabric_HiPCO0.03/SF, fabric_HiPCO0.05/SF, and fabric_HiPCO0.06/SF, and those with a crystallized SF top layer were labeled as fabric_HiPCO0.01/SFcr, fabric_HiPCO0.03/SFcr, fabric_HiPCO0.05/SFcr, and fabric_HiPCO0.06/SFcr (detail in Table 1).

**Figure 1:**
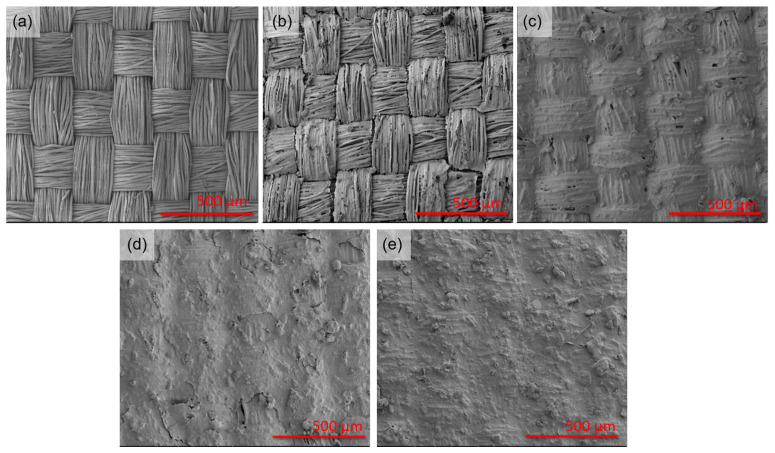
SEM images of silk fabric (**a**), and silk fabric functionalized with HiPCO of different concentrations: 0.01%wt. (**b**), 0.03%wt. (**c**), 0.05%wt. (**d**), and 0.06%wt. (**e**).

Figure 1 shows SEM images of the fabric before and after functionalization with HiPCO of different concentrations. SEM images were selected with magnifications that allow assessment of the functionalization process. With the increasing HiPCO concentration on the fabric (from 0.01 to 0.06%wt.), the silk weave and spaces between fibers become less apparent due to the high amount of HiPCO.

**Figure 2:**
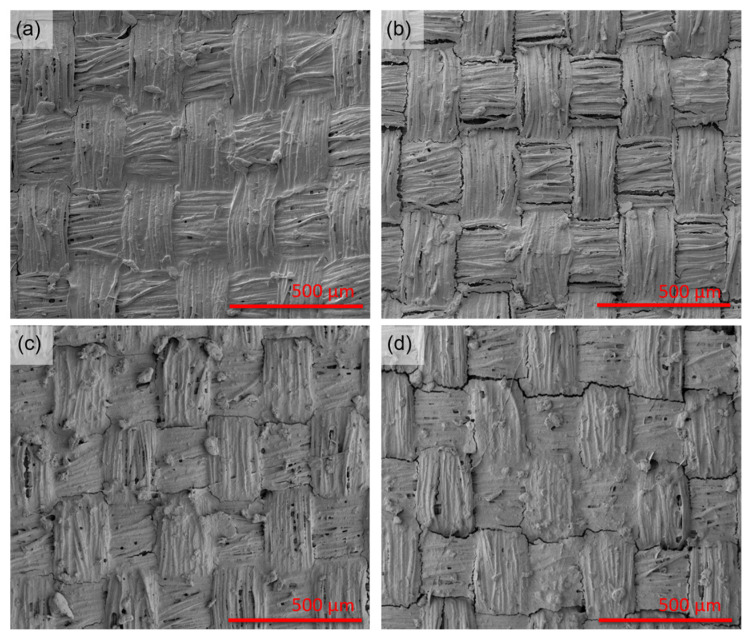
SEM images of silk fabric functionalized with 0.01%wt. (**a**,**b**), and 0.03%wt. (**c**,**d**) HiPCO, covered with SF (**a**–**d**), before (**a**,**c**) and after (**b**,**d**) crystallization process.

Figure 2 shows selected concentrations of HiPCO-functionalized fabric, further covered with SF before and after crystallization. The fabric with various concentrations of HiPCO was covered with the same amount of SF (4.8 g/m^2^). Figure 2a,c reveal HiPCO-functionalized fabric covered with SF, which does not differ significantly after the crystallization process (Figure 2b,d). The samples after the crystallization process may show slight cracks, which also prove that SF was crystallized and took a dominant β-sheet form. The β-sheets formed during crystallization are stacked to form tightly packed β-crystallites, folding into an ordered, repeating structure of antiparallel β-sheets. The folding process can cause the formation of some cracks within the layer. Further research in this study revealed that they do not significantly influence the protective properties of SF coverage and still support HiPCO on the fabric.

**Figure 3:**
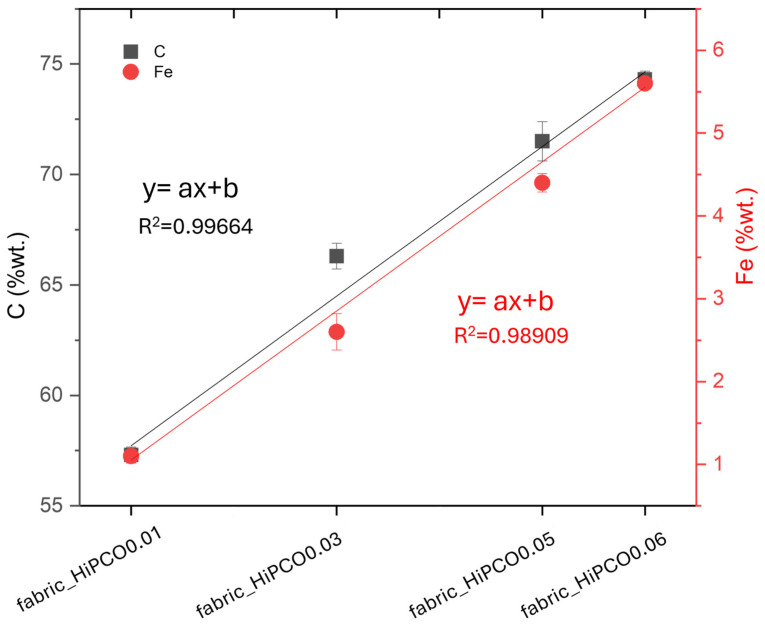
The weight percentage of C and Fe in silk fabric samples functionalized with HiPCO of different concentrations.

EDS analysis reveals the main elements related to silk fabric and HiPCO (Table 2). The elements characteristic of silk fibroin amino acids, such as carbon, nitrogen, and oxygen, are present in all samples. Moreover, all samples contain trace amounts of sodium and sulfur. Sodium remains in silk fabric due to the application of sodium carbonate for the degumming process in the first stages of fabric production [20]. Silk fabric consists of silk fibroin protein composed of heavy (H, ~390 kDa) and light (L, ~26 kDa) chains linked together via a single disulfide bond [21]. The presence of sulfur in the fabric indicates natural silk. However, sodium was also present in the surfactant used for HiPCO dispersion (bile salt–cholic acid–deoxycholic acid–sodium salt mixture); its amount does not increase with the increase in HiPCO content. It demonstrates the effectiveness of water rinsing, after HiPCO deposition, in removing the surfactant from the samples. Moreover, for samples functionalized with HiPCO, another element, iron, is present due to the presence of the metal catalyst in the nanotube synthesis process. The concentration of iron and carbon, which are the main nanotube elements, increases with increasing HiPCO concentration on the fabric (Figure 3).

**Figure 4:**
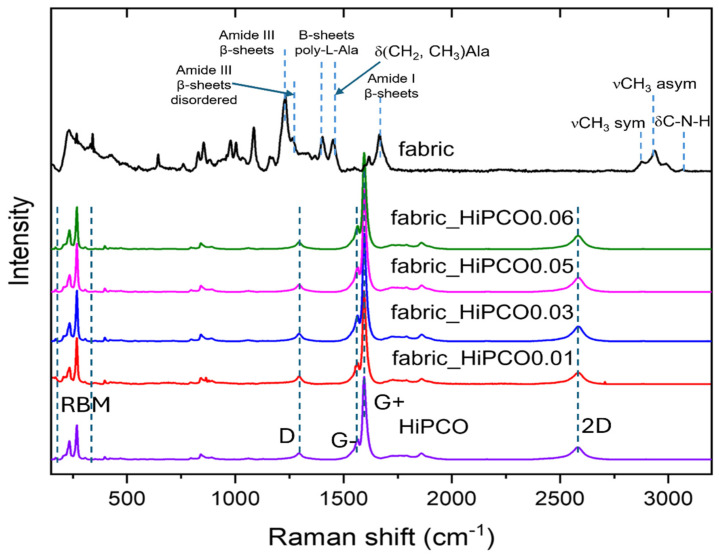
Raman spectra of silk fabric, HiPCO, and silk fabric functionalized with HiPCO of different concentrations.

The Raman spectrum of HiPCO presents characteristic bands for these CWNTs [22,23], which are present for all silk fabric samples functionalized with HiPCO (Figure 4). In the low frequency range of 100 to 300 cm^−1^ there are radial breathing mode (RBM) bands characteristic of SWNTs and related to radial vibrations of nanotubes. Two dominant bands at 1596 and 1564 cm^−1^ are correlated with G+ and G-, responsible for vibrations along and perpendicular to the tube axis. A low-intensity D (disorder) band at 1293 cm^−1^ indicates a small number of defects. The band at 2582 cm^−1^ is called 2D and is related to D-band overtone. Typical Raman bands for silk fabric are clearly visible only for pure fabric, because the signal intensity of SWNTs excites silk fibroin from the fabric. Raman bands characteristic of silk fabric are present at the same number and position for silk fabric after functionalization with HiPCO, but their intensity is significantly lower. Typical silk fibroin amide bands are noted, amide I at 1664 cm^−1^, indicating a β-sheet stable structure of the protein. Amide III bands at 1230 cm^−1^ also revealed a β-sheet ordered structure of fibroin with a small contribution of β-sheet disordered with a band at 1264 cm^−1^ [20]. The two bands at 1402 and 1452 cm^−1^ are related to vibrations of one of the most important silk fibroin amino acids, alanine (Ala). The band at 1402 cm^−1^ originates from β-sheet parts of poly-L-alanine [24] and 1452 cm^−1^ corresponds to CH_2_ and CH_3_ bending in alanine [25].

**Figure 5:**
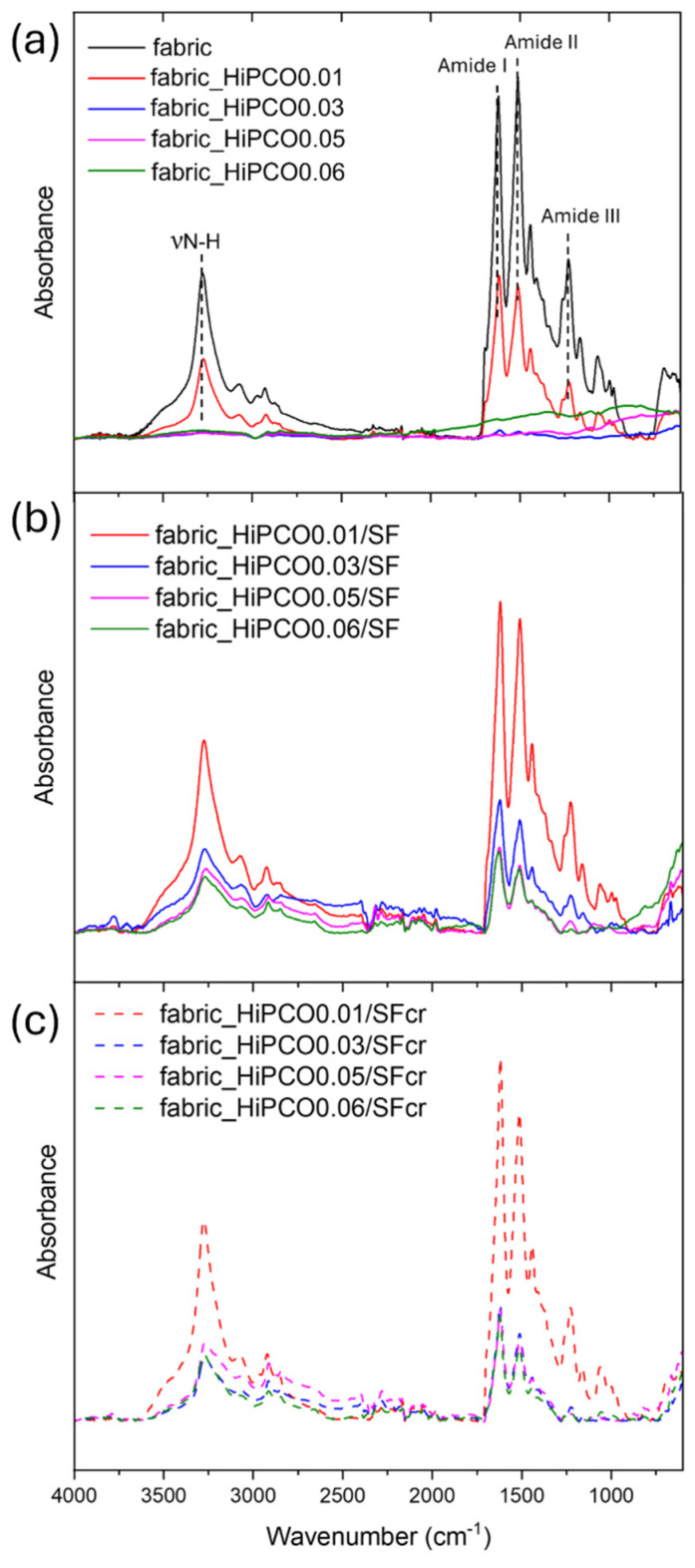
Infrared spectra of silk fabric and silk fabric functionalized with HiPCO of different concentrations (**a**), functionalized silk fabrics with HiPCO and coated with silk fibroin (**b**), functionalized silk fabrics with HiPCO, coated with silk fibroin and after crystallization process of SF (**c**).

The infrared spectroscopy studies were applied as a valuable tool for the evaluation of silk fibroin secondary structure and a complementary analysis to Raman spectroscopy. The main bands typical for silk fibroin were noted at 1622, 1512, and 1262 cm^−1^ indicating β-sheets conformation of the protein amide I, II, and III, respectively (Figure 5a) [26]. The random coil structure is also present due to the presence of the band of amide III at 1228 cm^−1^. The broad band at 3274 cm^−1^ indicates stretching vibrations of nitrogen–hydrogen bonds. All above mentioned bands characteristic for silk fibroin are present for non-functionalized silk fabric and for the fabric functionalized with the lowest concentration of HiPCO (fabric_HiPCO0.01), since higher concentration of nanotubes covers the absorbance signal from the fabric (Figure 5a). After covering the HiPCO-functionalized fabric samples with the protein, HiPCO are between silk fibroin on one side, in the form of silk fabric, and on the other side protein itself, as a coating. It results in typical SF bands appearing in FTIR spectra for all samples, with the most intense absorbance for fabric_HiPCO0.01 because the lowest concentration of nanotubes allows collected absorbance not only from SF coating but also from silk fabric (Figure 5b). After the crystallization process, typical bands for SF are also noticed (Figure 5c), indicating the presence of SF coating.

**Figure 6:**
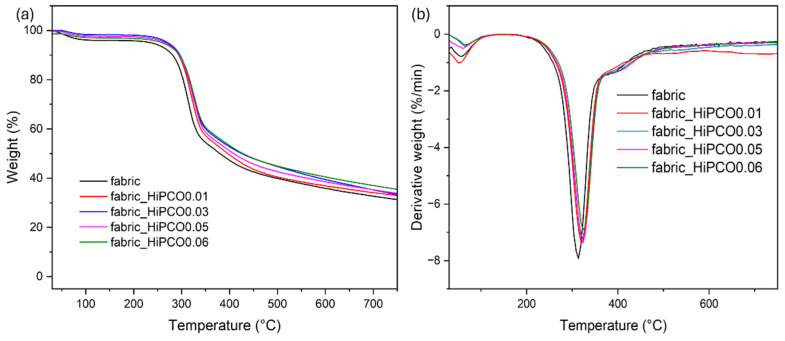
TG (**a**) and DTG (**b**) thermograms of silk fabric and silk fabric functionalized with HiPCO of different concentrations.

Figure 6 shows the thermograms of silk fabric before and after the functionalization with HiPCO. The main thermal decomposition peak is at 313.4 °C and is related to the thermal decomposition of silk fibroin [27]. For all functionalized samples with SWNTs, the maximum of the thermal decomposition band is shifted to higher temperatures, to 317.3 °C, 323.4 °C, 323.5 °C, 323.8 °C, and 325 °C, respectively, for 0.01, 0.03, 0.05, and 0.06%wt. of HiPCO. The thermal decomposition process also starts at a higher temperature compared to the onset temperature of the non-functionalized silk fabric, with onset temperature of 288.6 °C, 297.0 °C, 292.9 °C, 300.0 °C, and 302.0 °C, respectively, for 0.01%, 0.03%, 0.05%, and 0.06% wt. of HiPCO. After the functionalization process, the HiPCO samples revealed slightly higher resistance to heat compared to the pure silk fabric. HiPCO functionalization increases the thermal resistance of silk fabric slightly.

SWCNTs can increase the thermal resistance of textiles by forming a functional coating or network on fabric fibers, which can reduce air permeability and trap heat. SWCNTs can be used in a coating on flame-retardant fabrics to improve their thermal comfort by reducing heat transmission [28].

**Figure 7:**
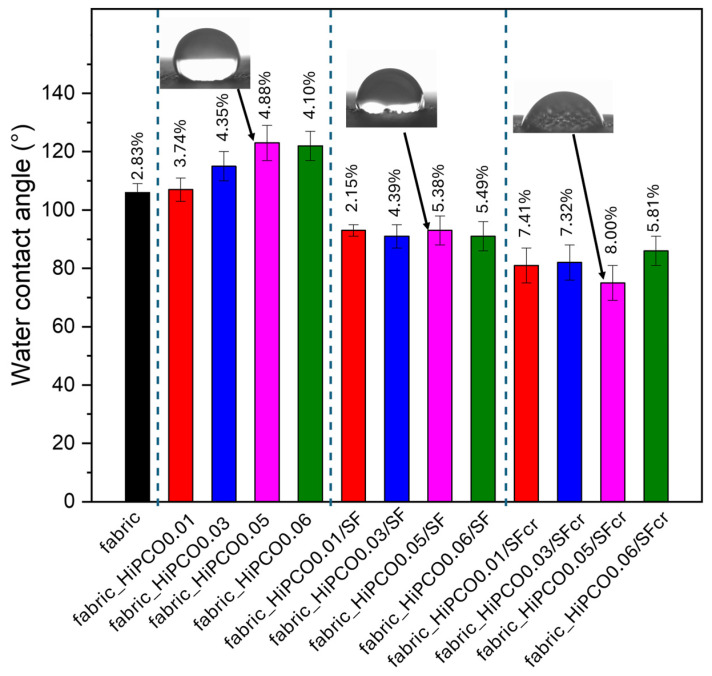
Water contact angle of silk fabric and silk fabric functionalized with HiPCO of different concentrations, further coated with silk fibroin, and crystallized. CV (coefficient of variation) values were provided for each group of analyzed samples. Insets: The examples of water drops on the fabric after the subsequent stages of the functionalization process.

The water contact angle for pure silk fabric was about 106 ± 3° (Figure 7). It is typical for silk fabric to be characterized by the parameters described below (Section 3) [12].

SWNTs are a highly hydrophobic material, and due to strong van der Waals interactions between sp^2^-sp^2^-hybridized carbons, they aggregate in nearly all solvents [29]. Water contact angles increase slightly after the functionalization with HiPCO, and the fabric becomes more hydrophobic (Figure 7). This is also the result of efficiently removing bile salt surfactant. After coating the samples with silk fibroin, the water contact angle decreases to about 90° due to the hydrophilic nature of the SF layer [30]. The crystallization process caused the water contact angle to decrease slightly, even though the SF layer became water-insoluble. The key issue of this phenomenon is the structure of silk fibroin. B. mori crystallized silk fibroin has a dominant β-sheet structure and mostly hydrophobic nature [31], with charged hydrophilic terminal regions that end-cap a hydrophobic core consisting of repeating sequences of glycine, alanine, and serine residues [32]. SF crystals, which consist of “stacked” β-sheets with hydrophobic interactions between amino acid side chains, are embedded in a hydrophilic amorphous matrix [33]. The structure of SF and the content and arrangement of both crystalline and amorphous parts are crucial for the wettability of SF-coated surfaces. Most β-sheets domains tightly cover the coated surface, and the amorphous, hydrophilic parts are exposed externally, which makes the surface more hydrophilic.

**Figure 8:**
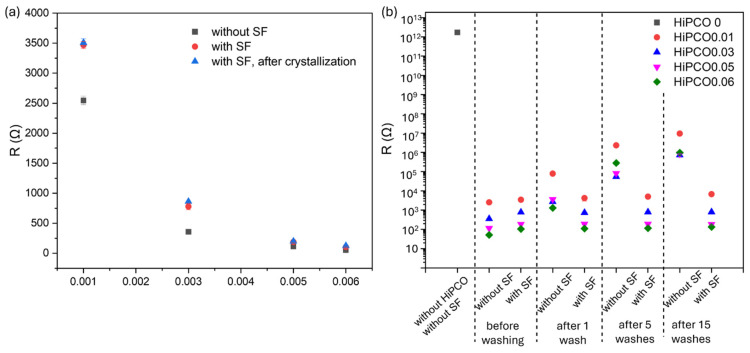
Resistance of (**a**) silk fabric coated with HiPCO, and after silk fibroin coverage and further SF crystallization. (**b**) Resistances of non-functionalized silk fabric and functionalized with HiPCO, and with HiPCO and SF, before washing and after 1, 5, and 15 wash cycles.

Figure 8a presents the resistance value depending on HiPCO content on the fabric. Resistance decreases with increasing HiPCO content from about 2.5 × 10^3^ Ω for 0.01%wt. to 50 Ω for 0.06%wt. of HiPCO. This means that the higher the SWNT deposition, the higher the electrical conductivity of the fabric. It was reported that HiPCO incorporated through the dying approach allowed the obtaining of conductive textiles [34]. After coating HiPCO-functionalized fabric with silk fibroin, the resistance increases slightly for the lowest HiPCO deposition on the fabric. In case of highest HiPCO deposition of 0.05 and 0.06%wt., the amount of nanostructures provides developed percolation paths and facilitates electron flow. The SF coating does not affect conductivity for the sample with higher deposition of HiPCO. Moreover, the crystallization process of SF does not affect the conductivity of the fabric. The resistance values for the samples coated with SF before and after the crystallization are similar (Figure 8a).

The influence of SF coating was evaluated by resistance measurements after washing (Figure 8b). The resistance of the fabric before functionalization with HiPCO is about 1.7 × 10^12^ Ω. The resistance of HiPCO-functionalized fabric decreases significantly, and when covered with SF, it does not change significantly even after 15 wash cycles due to the protective influence of the crystallized SF coating, which protects HiPCO against washing out. The resistance of the fabric without the SF coating increases significantly after just one wash cycle and constantly increases after subsequent wash cycles (Figure 8b). SF coating can be stated as an efficient protective coating for designing wearable electronics due to (i) the selection of a natural, biocompatible, and non-toxic product; and (ii) making it insoluble in water in the crystallization process, which does not affect the fabric and conductive nanostructures because it is carried out in a gas phase. SF coating is a crucial element for realizing further smart textile applications, enabling the development of reusable devices.

## 3. Materials and Methods

### 3.1. Functionalization of Silk Fabric with HiPCO and Further with Silk Fibroin Layer

Silk fabric applied to the study is characterized by a thickness of 0.2 mm, a mass per unit area of 70 g/m^2^, and linear mass of weft and warp yarns of 37 dtex. Silk fabric was functionalized with single-wall carbon nanotubes (HiPCO-SWNTs-Purified, HS 28030), purchased from NanoIntegris, Boisbriand, QC, Canada. SWNTs were dispersed in an aqueous solution of bile salt (Merck, Warsaw, Poland) 1%wt. at a concentration of 0.1 mg/mL. Silk fabric was impregnated with SWNTs solutions of different volumes to obtain different HiPCO deposition. Then the silk fabric was vigorously rinsed in water five times to remove bile salt. Silk fabric with HiPCO was heated for 10 min at 100 °C.

To improve the stability, durability, and washability of the silk fabric with HiPCO, the samples were coated with silk fibroin (SF). Silk fibroin was sourced from *Bombyx mori* cocoons (Shenzhen, China). The cocoons were cut into smaller pieces, and 0.4 g of cocoon material was immersed in 200 mL of 0.02 M sodium carbonate (Na_2_CO_3_, Merck, Warsaw, Poland) aqueous solution. They were stirred and heated at 100 °C to effectively carry out the degumming process. After removing silk sericin from the cocoons, the silk fibroin was rinsed three times in distilled water and dried for 24 h at 40 °C. Next, the silk fibroin was dissolved in an aqueous solution of anhydrous calcium chloride (CaCl_2_, Merck, Warsaw, Poland) and ethanol at 75 °C for 5 min. The molar ratio of CaCl_2_:H_2_O:EtOH (99.8%, Avantor, Gliwice, Poland) was 1:8:2. After dissolving SF and cooling to room temperature, the solution was dialyzed in a 12 kDa dialysis bag with a volume of 20 mL (Merck, Warsaw, Poland). The dialysate water (1 L) was changed eight times over three days. Then, 1 mL of SF solution was dried and weighed to determine its concentration, which was 30 mg/mL.

SF at the concentration of 30 mg/mL was cast on SWNT modified silk fabric samples (0.16 L to m^2^) at 21 °C and HR 35 ± 2%. Then, the samples were dried at room temperature for 24 h, and SF was crystallized to make it insoluble in water. The samples were treated with ethanol (99.8%, Avantor, Gliwice, Poland) vapor for 3 days at 21 °C.

### 3.2. Characterization

The sample surface was analyzed using scanning electron microscopy (Quanta 200 (FEI, Company (Field Electron and Ion Company), Hillsboro, OR, USA)) at an accelerating voltage of 5 kV. Prior to the SEM measurements, the samples were covered with a 3 nm gold layer. The chemical composition was analyzed using energy dispersive spectroscopy (EDS, INCA Energy X-ray energy dispersion spectrometer, Oxford Instruments, Abingdon, UK) from an area of 1.175 mm^2^ of the samples that were not covered in gold, at an accelerating voltage of 20 kV. The statistical evaluation of the EDS measurements was carried out with the INCA energy software 450 (version 4.15).

The samples were studied using Fourier Transformed Infrared (FTIR) and Raman spectroscopies. FTIR absorption spectra were recorded using a BRUKER Vertex 70 FTIR spectrometer (Bruker, Bremen, Germany) with a diamond ATR Golden Gate adapter. The measurements were carried out in the spectral range of 600 to 4000 cm^−1^, with a resolution of 4 cm^−1^. Raman spectra were obtained using an inVia Renishaw Raman Microscopy System (Renishaw, Wotton-under-Edge, UK) and an applied 20× microscope objective (LEICA, Wetzlar, Germany). Each Raman spectrum was obtained in the range of 150 to 3200 cm^−1^, with five accumulations, corrected by the WIRETM 5.3 software. The excitation source was a 785 nm laser. FTIR and Raman spectra were collected in at least three replicates for each sample.

The thermogravimetry (TG, 209F1 Libra, Netsch, Netzsch, Selb, Germany) measurements were performed in the temperature range of 30–750 °C, with a heating rate of 10 °C, under a nitrogen flow of 25 mL min^−1^. Prior to measurements, the samples were weighed to be about 4 mg and placed in a ceramic crucible with a volume of 85 µL. Derivative thermogravimetry (DTG) results were also analyzed.

The wettability of silk fabric before and after the functionalization was studied by measurements of water contact angle using a goniometer (PGX Fibro System AB, Stockholm, Sweden) in a dynamic mode. Deionized water was applied to the analyzed surface at a constant volume of 4 (±0.2) µL.

The electrical resistance of the sample was measured using a 6206 teraohmmeter (ELTEX) at a temperature of 23 (±2) °C and relative humidity of 25 (±5)%. The samples for electrical resistance measurements were prepared for further measurements with two electrodes, which were in the form of a ring with a diameter of 15.5 mm, made of brass-plated steel. The distance between the electrodes was 10 mm, and they were placed on the fabric just before the measurements at the same place. The places for the electrodes were covered with silver paint (Leitsilber L200N, Plano GmbH, Wetzlar, Germany).

The resistance of the samples was measured before and after silk fibroin coating to study the influence of SF coating on their efficiency and reusability. They were washed according to the international standard [35] for 30 min at 30 °C once, five, and fifteen times in a mechanical device containing a rotatable shaft which supports radially, with stainless steel containers with a diameter of about 75 mm, a height of about 125 mm, and a capacity of about 550 mL (Linitest, Original Hanau, Hanau, Germany). The containers are rotated at a frequency of about 40 min^−1^. The temperature of the water bath was thermostatically controlled to maintain the test solution at 40 ± 2 °C. The applied detergent was WOB (without optical brightener, AATCC (American Association of Textile Chemists and Colorists) 1993 Standard Reference Detergent WOB, properties and composition given in table 2 in the standard [35]), and the water was Grade 3 (complying with ISO 3696 [36]). After washing, the samples were rinsed twice in two separate 100 mL portions of water at 40 °C and then dried by hanging them in the air at a temperature not exceeding 60 °C (Heraeus Function Line UT6P Programmable Drying Oven, Akribis Scientific Supplies Ltd., London, UK).

## 4. Conclusions

The surface coating with silk fibroin, presented in this report, was proposed as a way of protecting textile wearable electronics with a non-toxic material. Silk fabric functionalized with HiPCO SWNTs was selected as a model conductive textile structure. HiPCO makes silk fabric electroconductive. The resistance of the fabric decreases from the level of 10^12^ Ω to 10^4^, 10^3^, and 10^2^ Ω, respectively, for 0.01, 0.03, 0.05, and 0.06%wt. of HiPCO on the fabric. HiPCO were directly deposited on silk fabric, without applying any procedure to ensure they are bound to the silk fabric surface, and they are not stable on the fabric. To protect HiPCO against external conditions and washing out, as well as make HiPCO-functionalized fabric washable and reusable, they were covered with SF. Then the SF coverage was crystallized by ethanol vapor to make the protein protective coating insoluble in water. SEM and FTIR analyses confirmed the presence of SF coating, and the wettability of the silk fabric surface was changed after the modification with HiPCO, SF, and after the crystallization process.

The resistance of HiPCO-functionalized silk fabric after coverage with SF and the crystallization process does not change significantly, even after 15 wash cycles.

Our findings indicate that textile electronic structures covered with SF represent a very promising building block for the fabrication of effective and non-toxic wearable devices in the near future. It will enable the design of devices for continuous health monitoring, personalized treatments, real-time diagnostics, and drug delivery systems.

## Figures and Tables

**Table 1:** Samples names and descriptions of silk fabric functionalized with HiPCO and further covered with SF.

Sample Name	Sample Description	HiPCO SWNT on Silk Fabric (%wt.)	HiPCO SWNT Deposition on Silk Fabric (mg/m^2^)	Silk Fibroin Deposition (g/m^2^)
**fabric**	silk fabric non-functionalized	**-**	**-**	**-**
**HIPCO**	non-modyfied HiPCO SWNTs	**-**	**-**	**-**
**fabric_HIPCO0.01**	silk fabric with 0.01%wt. of HiPCO	0.011 ± 0.003	80 ± 4	**-**
**fabric_HIPCO0.03**	silk fabric with 0.03%wt. of HiPCO	0.032 ± 0.005	160 ± 11	**-**
**fabric_HIPCO0.05**	silk fabric with 0.05%wt. of HiPCO	0.052 ± 0.003	280 ± 15	**-**
**fabric_HIPCO0.06**	silk fabric with 0.06%wt. of HiPCO	0.062 ± 0.003	400 ± 14	**-**
**fabric_HIPCO0.01/SF**	silk fabric with 0.01%wt. of HiPCO and covered with 4.8 g/m^2^ SF	0.011 ± 0.003	80 ± 4	4.8 ± 0.2
**fabric_HIPCO0.03/SF**	silk fabric with 0.03%wt. of HiPCO and covered with 4.8 g/m^2^ SF	0.032 ± 0.005	160 ± 11	4.8 ± 0.4
**fabric_HIPCO0.05/SF**	silk fabric with 0.05%wt. of HiPCO and covered with 4.8 g/m^2^ SF	0.052 ± 0.003	280 ± 15	4.8 ± 0.2
**fabric_HIPCO0.06/SF**	silk fabric with 0.06%wt. of HiPCO and covered with 4.8 g/m^2^ SF	0.062 ± 0.003	400 ± 14	4.8 ± 0.5
**fabric _HIPCO0.01/SF**cr	silk fabric with 0.01%wt. of HiPCO and covered with 4.8 g/m^2^ SF, crystallization	0.011 ± 0.003	80 ± 4	4.8 ± 0.2
**fabric _HIPCO0.03/SF**cr	silk fabric with 0.03%wt. of HiPCO and covered with 4.8 g/m^2^ SF, crystallization	0.032 ± 0.005	160 ± 11	4.8 ± 0.4
**fabric _HIPCO0.05/SF**cr	silk fabric with 0.05%wt. of HiPCO and covered with 4.8 g/m^2^ SF, crystallization	0.052 ± 0.003	280 ± 15	4.8 ± 0.2
**fabric _HIPCO0.06/SF**cr	silk fabric with 0.06%wt. of HiPCO and covered with 4.8 g/m^2^ SF, crystallization	0.062 ± 0.003	400 ± 14	4.8 ± 0.5

**Table 2:** Results of EDS analysis of non-functionalized silk fabric, silk fabric functionalized with HiPCO of different concentrations.

	C (%wt.)	N (%wt.)	O (%wt.)	Na (%wt.)	S (%wt.)	Fe (%wt.)
**fabric**	48.1	21.2	30.3	0.3	0.1	**-**
**fabric_HiPCO0.01**	57.3	14.7	26.6	0.2	0.1	1.1
**fabric_HiPCO0.03**	66.3	8.6	22.3	0.1	0.1	2.6
**fabric_HiPCO0.05**	71.5	6.2	17.7	0.1	0.1	4.4
**fabric_HiPCO0.06**	74.4	4.3	15.5	0.1	0.1	5.6

## Data Availability

The data presented in this study are available on request from the corresponding author.
